# Refractory symptoms and end of life midazolam use in cancer patients, a single center experience

**DOI:** 10.1017/S1478951525100461

**Published:** 2025-08-08

**Authors:** Anna-Maria Tolppanen, Annamarja Lamminmäki, Enni Järvenpää, Vesa Kataja, Kristiina Tyynelä-Korhonen

**Affiliations:** 1 Center of Oncology, The Wellbeing Services County of North Savo, Kuopio, Finland; 2 University of Eastern Finland, Kuopio, Finland; 3 Department of Diagnostic Services, Wellbeing Services County of Central Finland, Jyväskylä, Finland; 4 Oncology Clinic, Wellbeing Services County of South Savo, Mikkeli, Finland; 5 The Palliative Care Center, Päijät-Häme Wellbeing Services County, Lahti, Finland

**Keywords:** Refractory symptoms, Midazolam, Palliative sedation, Palliative care, Cancer

## Abstract

**Objectives:**

Cancer patients often suffer from refractory symptoms near death. The use of sedatives aims to relieve suffering caused by these symptoms. The practice varies broadly. The aim of this study was to evaluate the role and trends of midazolam use in cancer patients dying in a university hospital oncology ward.

**Methods:**

The study population of this retrospective registry-based study consists of patients who died in a university hospital oncology ward in Eastern Finland in 2010–2018 (n = 639). Information about treatment decisions, midazolam use, and background factors were gathered.

**Results:**

During the study period, 14.7 % of the patients dying in the ward received midazolam with sedative intent prior to death. 4.7 % (n = 30) of the whole study population had continuous infusion and the rest of the midazolam use was one or multiple single doses. Documented discussion of possible palliative sedation (PS) use was found in almost one third of all patients. Out of those, eventually receiving midazolam with sedative intent, two thirds had had this discussion. The most common symptoms leading to midazolam were dyspnea, pain, and delirium. In continuous use the median midazolam infusion rate was 4.0 mg/h. The continuous infusion started median of 23.25 h and multiple single doses 19 h before death. If only one dose of midazolam was needed, it was given median of 30 minutes prior to death and the most common symptom was dyspnea. Those who received midazolam were more likely to be younger (*p = 0.003*) and had had a palliative outpatient clinic visit (*p = 0.045*).

**Significance of results:**

This is the first study to report the trends and practices of midazolam use for refractory symptoms in Finland. Midazolam was used for approximately every 7^th^ dying cancer patient. Applying midazolam was supported by a history of palliative clinic visits and younger age.

## Introduction

Patients with advanced cancer often suffer more physical and psychological symptoms as the disease progresses towards the end of life. The symptoms may become intolerable, and if not adequately relieved, may become difficult to manage, i.e. refractory (Barbera et al. 2010; Cherny 2014; Conill et al. 1997; *The role of Palliative Sedation in palliative care. eBook edited by Ian Koper, Jeroen Hasselaar and Cathy Payne.* 2024). At the end of life, the most common cancer related symptoms are dyspnea, pain, anxiety, and delirium (Teunissen et al. 2007).

Worldwide, there is a lot of variance in the use of sedative agents with in the end-of-life (EOL) care of patients with refractory symptoms (Abarshi et al. 2017; Cherny 2014; Gurschick et al. 2015). According to the EAPC (Cherny and Radbruch 2009), ESMO (Cherny 2014) and American Academy of Hospice and Palliative Medicine guidelines (‘Statement on Palliative Sedation Approved by the AAHPM Board of Directors on December 5 2014’), palliative sedation (PS) is the monitored use of medications with the intent to achieve a state of decreased or absent awareness in terminally ill patients. Itʹs a measure of last resort with the aim to relieve unbearable suffering due to severe and refractory symptoms at the end of life (Cherny 2014; Cherny and Radbruch 2009; ‘Statement on Palliative Sedation Approved by the AAHPM Board of Directors on December 5 2014’). In most cases PS is used for the treatment of pain, dyspnea, agitated delirium, and convulsions (Cherny 2014). It can be delivered continuously or intermittently until death (*The role of Palliative Sedation in palliative care. eBook edited by Ian Koper, Jeroen Hasselaar and Cathy Payne.* 2024). PS should only be considered if the prognosis is in the range of hours or days. The level of sedation should be the lowest possible, but to provide sufficient symptom control and relieve of suffering (Cherny and Radbruch 2009). There is evidence that PS doesn’t have an effect on patient survival (Maeda et al. 2016; Maltoni et al. 2012; Maltoni and Setola 2015).

PS is estimated to precede 10–18 % of all deaths in Europe (Payne and Hasselaar 2020). There is data from the use of PS in several European countries (*The role of Palliative Sedation in palliative care. eBook edited by Ian Koper, Jeroen Hasselaar and Cathy Payne.* 2024). Little is known about the use of sedatives at EOL care in Northern Europe and to our knowledge the only data published from Finland is about the subcutaneous use of dexmedetomidine (Uusalo et al. 2018) and the attitudes of nurses regarding PS (Heino et al. 2021).

Many studies about sedatives at EOL care contain only continuous use of sedative agents and in these studies the definition of PS is a continuous infusion (Miccinesi et al. 2006; Prado et al. 2018). The most often used sedative worldwide is midazolam (*The role of Palliative Sedation in palliative care. eBook edited by Ian Koper, Jeroen Hasselaar and Cathy Payne.* 2024). In real life, the use of midazolam with sedative intent may be much broader at EOL care. The aim of this study was to evaluate the role and practices of midazolam use with sedative intent in cancer patients dying in a Finnish university hospital oncology ward.

## Methods

The study was designed as a retrospective registry-based study.

### Study setting and cohort selection

The population of this study consists of patients who died in Kuopio University Hospital (KUH) oncology ward between January 1^st^ 2010 and December 31^st^ 2018, excluding patients who died in November or December 2011. The original study design excluded patients who died during these two months during which the palliative outpatient clinic was established, and the logistics was changing and affecting the functioning.

There were 639 patients (58.1 % males, n = 371, and 41.9 % females, n = 268) included in the study. Most patients had solid tumors, since at that time almost all lymphomas were treated at the Hematology Unit of KUH.

The Finnish tax-financed national health care service system provides cancer care for all residents with minimal cost to the patients. KUH is the main hospital in the Health Care District of North-Savo and it is responsible for cancer care for some 247,000 residents in its catchment area. During the study period, the KUH oncology ward had a capacity of 38 beds until the years 2011–2012 when it was reduced to 18 beds. Most of the patients were referred to the ward from the Emergency Department (ER), others from oncology or palliative outpatient clinics, while some had come for scheduled treatment.

Palliative care cancer patients are admitted to the oncology ward (attending physician being an oncologist) when they need inpatient care since there is no separate palliative care ward in KUH. At that time, there was no palliative care specialists in the ward. Practically across the street from the hospital, there is a hospice run by the city of Kuopio, while basic level palliative and end-of-life care is provided by the wards of other municipal primary health care centers.

## Methods

In KUH all patient data is in electronic format. The information about sedatives and background factors (including cancer diagnosis, age, and treatment decisions) were retrieved from the records. The researchers (authors AT, KTK and EJ) reviewed all medical records. In case the patient had more than one cancer, only the cancer the patient was being treated for and dying to at the time was included in the final analyses.

The information about the use of midazolam for sedative purposes at the end of life (EOL) was recorded. We excluded other sedative agents as in the majority of the cases, midazolam was used. The use of midazolam e.g., before some diagnostic examination (for example endoscopy) or symptom relief with no clear intent to provide palliative sedation was excluded. If a single dose of midazolam was given with a clear intent to sedation at EOL, it was captured. The information about the type of midazolam use was gathered (continuous, only one dose or multiple single doses), also the starting time of midazolam, and the doses were documented. The beginning of midazolam use was recorded to the nearest 15 minutes. In addition, the information whether there was a record of a conversation about the use of PS beforehand was gathered. The dates of palliative and also EOL care decisions were retrieved. The EOL care decision is widely used in Finland to define the ultimate dying phase of the illness, usually covering the last few days or weeks of life. Customarily, during that time antibiotics aren’t used, no blood tests are taken, no scans are made etc.; i.e., the focus is only to treat symptoms and relieve suffering.

### Statistical analysis

All statistical analyses were performed with IBM SPSS 27 software. The Chi-square test was used to test the difference between categorical variables, and the T-test was used to investigate the difference in age between groups.

### Ethical aspects

This retrospective registry-based study was permitted by the KUH administration authority. The North-Savo Health Care District Ethics Committee performed an evaluation and approved the study.

## Results

In the whole population, the most common cancer types were lung, breast, colorectal and, pancreatic cancer. Out of these, lung cancer was the most common cancer diagnosis both in the whole population and in patients receiving midazolam, the second most common being colorectal cancer. The group “other” consisted of all the other cancer types (Supplementary Table 1). The proportion of prostate cancer was 4.1 % (n = 26) and of lymphomas only 1.6 % (n = 10). In 88.3 % of the patients, the diagnosis of cancer was biopsy-based and pathologically verified, while the rest had a clinical diagnosis based e.g., on radiology findings. A second malignancy had been diagnosed for 8.9 % (n = 57) of the patients; most often it was colorectal, prostate, or breast cancer.

**Figure 1.**
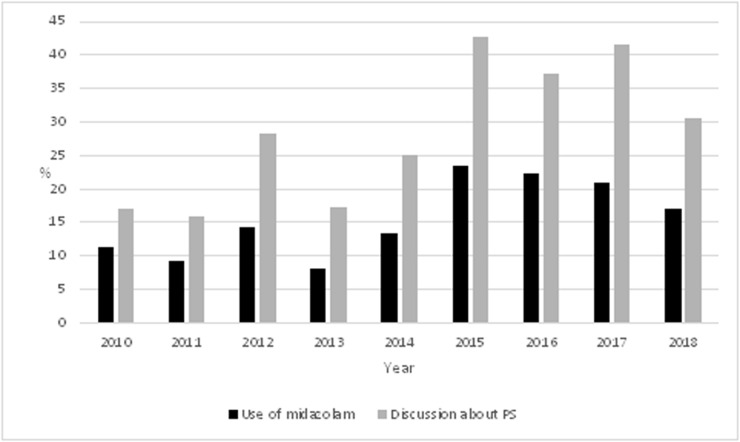
The use of midazolam with sedative intent and discussions, yearly alternations.

During the nine-year study period, altogether 14.7 % (n = 94) of the patients received midazolam with sedative intent prior to death. There was variation considering individual years; in 2013 only 8.0 % (n = 7) of patients had midazolam while in 2015 the proportion was 23.3 % (n = 17) (Fig. 1). In the whole study population (n = 639), the documentation of discussion about the possible use of PS in the patient records could be found in 26.8 % (n = 171) of the cases. Out of these 38.0 % (n = 65) eventually had midazolam with sedative intent. Additionally, there were 29 patients who needed midazolam, but there was no record of a conversation about possible PS. In 39.9 % (n = 255) of the patients there was a PS drug added in the list of medicines in case there would be a need to start PS. There was no monitoring the depth of sedation with specific scales in any of the patients.

**Figure 2.**
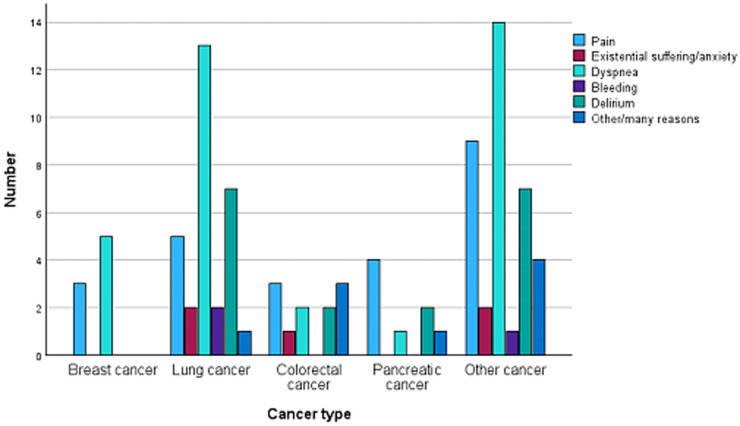
Symptoms leading to the use of midazolam with sedative intent by cancer type.

The key characteristics of patients who received midazolam are presented in Table 1. DNAR was made in 74.5 % of the patients, but the percentage changed markedly over the years. In 2010 0 % had DNAR, 2011 14.3 %, and 2012 50 %, and after 2013 it was close to 100 %. Palliative and/or EOL care decision was made in 88.3 % of the patients. The main reasons for admissions were deteriorating general condition, pain, and dyspnea. The most common principal refractory symptoms which required midazolam were dyspnea, pain, and delirium. Dyspnea was especially seen in lung cancer patients (Fig. 2). Midazolam was mainly administered intravenously or subcutaneously. The median inpatient days within six months prior to death was 23 days with those who had midazolam and 17 days with those who didn’t need it at EOL.

**Table 1.** Baseline characteristics of patients who had midazolam with sedative intent prior to death (n = 94)

Patient characteristics	median (range) n (%)
Age, years	62 (23 − 94)
Male	52 (55.3 %)
DNAR	70 (74.5 %)
Palliative and/or EOL care decision made	83 (88.3 %)
Cancer type	
Lung	30 (31.9%)
Colorectal	11 (11.7%)
Pancreas	8 (8.5%)
Breast	8 (8.5 %)
Other	37 (39.4%)
Reason for admission	
Pain	24 (25.5 %)
Deteriorating of general condition	22 (23.4 %)
Dyspnea	18 (19.1 %)
Bleeding	5 (5.3 %)
Delirium	4 (4.3 %)
Infection	4 (4.3 %)
Planned examination	4 (4.3 %)
Planned radiation therapy	3 (3.2%)
Planned oncological treatment	3 (3.2 %)
Other	7 (7.4 %)
Principal reason for midazolam use	
Dyspnea	35 (37.2 %)
Pain	24 (25.5 %)
Delirium	18 (19.1 %)
Existential suffering/anxiety	5 (5.3 %)
Bleeding	3 (3.2 %)
Other/many symptoms	9 (9.6 %)

**Figure 3.**
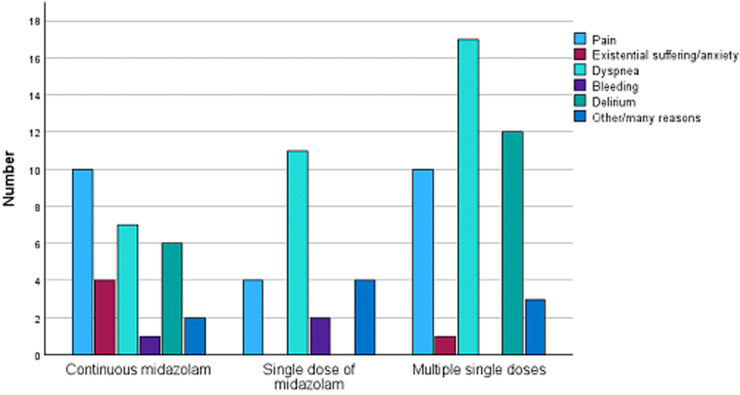
Refractory symptoms divided by midazolam use.

Median timing of midazolam usage was 15 h 30 min before death (Table 2). It was the longest in patients who had a continuous infusion (23 h 15 min). If two or more single doses were needed, the beginning was median of 19 h prior to death. If only a single dose of midazolam was needed, it was given just prior to death, and in those patients the most common refractory symptom was dyspnea (Fig. 3). If a continuous infusion was used, the median infusion dosage before death was 4.0 mg/h. The median dosage was 2.5 mg in those patients who needed only one dose of midazolam just prior to death. In cases with multiple doses, a median of 3 was needed and the combined median dosage was 10 mg (Table 2). Table 3 shows differences in patients with different types of midazolam usage.

**Table 2.** Type of midazolam with sedative intent

	median (range)
All patients with midazolam (n = 94)	
Beginning of midazolam	15 h 30 min (15 min – 194 h 15 min)
Continuous infusion (n = 30)	
Beginning	23 h 15 min (4 h 45 min − 194 h 15 min)
Dosage before death	4.0 mg/h (0.5 − 16.0 mg/h)
One dose of midazolam (n = 21)	
Beginning	30 min (15 min – 40 h)
Median dosage	2.5 mg (1–10 mg)
Two or more single doses (n = 43)	
Beginning	19 h (30 min – 190 h 45 min)
Number of doses	3 (2 − 30)
Midazolam dosage, all doses combined	10 mg (1.5–95 mg)

Compared to other patients, the patients who received midazolam were more likely to be younger and had had a palliative outpatient clinic visit (Table 4).

**Table 3.** Midazolam divided into continuous, one dose and multiple single doses

	Continuous (n = 30)	1 dose (n = 21)	Multiple single doses (n = 43)
	Median (range), n (%)	Median (range), n (%)	Median (range), n (%)
Age, years	59 (23 − 82)	67 (50 − 89)	62 (28 − 94)
DNAR	26 (86.7 %)	15 (71.4 %)	29 (67.4 %)
Palliative and/or EOL care decision made	28 (93.3 %)	16 (76.2 %)	39 (90.7 %)
Reason for midazolam usage			
Dyspnea	7 (23.3 %)	11 (52.4 %)	17 (39.5 %)
Pain	10 (33.3 %)	4 (19.0 %)	10 (23.3 %)
Delirium	6 (20.0 %)	0 (0.0 %)	12 (27.9 %)
Existential suffering/anxiety	4 (13.3 %)	0 (0.0 %)	1 (2.3 %)
Bleeding	1 (3.3 %)	2 (9.5 %)	0 (0.0 %)
Other	2 (6.7 %)	4 (19.0 %)	3 (7.0 %)

**Table 4.** Differences between patients with or without midazolam at EOL

	Midazolam with sedative intent		
	**No**	**Yes**	
	Mean (SD) n (%)	Mean (SD) n (%)	p-value for difference
All patients, n = 639	545 (85.3 %)	94 (14.7 %)	
Age, years	67 (10.9)	62 (14.1)	*0,006*
Gender			0,560
Male	319 (58.5 %)	52 (55.3 %)	
Female	226 (41.5 %)	42 (44.7 %)	
Palliative outpatient clinic visit			*0,045*
Yes	50 (9.2 %)	15 (16.0 %)	
No	495 (90.8 %)	79 (84.0 %)	
Palliative and/or EOL care decision made before death			0.062
Yes	437 (80.2%)	83 (88.3 %)	
No	108 (19.8 %)	11 (11.7 %)	
Cancer type			0.221
Lung	127 (23.3 %)	30 (31.9 %)	
Breast	85 (15.6 %)	8 (8.5 %)	
Colorectal	57 (10.5 %)	11 (11.7 %)	
Pancreas	59 (10.8 %)	8 (8.5 %)	
Other	217 (39.8 %)	37 (39.4 %)	

## Discussion

This retrospective data brings valuable information about the use of midazolam in end-of-life care in Finnish cancer patients. The use of midazolam in our ward was fairly common, but only 4.7 % of the whole study population received continuous infusion of midazolam. Patients who received midazolam were more likely to be younger than those who did not need it. They also had had a palliative outpatient clinic visit more often.

Worldwide, midazolam is the most commonly used sedative in EOL sedation (Alessia et al. 2022; Beller et al. 2015; Garetto et al. 2018; Schur et al. 2016). In a study conducted in several European countries, the use of sedation ranged from 2.5 % in Denmark up to 8.5 % in Italy (Miccinesi et al. 2006). In another study, applying PS varied between 7 % to 18 % of palliative care patients (Anquinet et al. 2012). In a review of 14 studies including mainly cancer patients (over 95 %) the proportion of PS ranged from 12 % to 67 % (Beller et al. 2015). In a multicenter Dutch study the prevalence of continuous PS was 27.8 % (Van Deijck et al. 2016b), whereas in a study by Pardo et al. (Prado et al. 2018) with cancer patients admitted at a tertiary cancer center the percentage was rather high, 54.2 %. There are signs indicating that the use of PS seems to be increasing (Bosshard et al. 2016; Rietjens et al. 2019). There is much variation and compared to literature, the use of midazolam with sedative intent in our ward was in line with earlier published data but at the lower level. Altogether, every 7^th^ patient had midazolam, the majority had single doses and continuous infusions were much less used. Midazolam use seemed to increase over the years in the KUH ward. Possible reasons may include changes in staff experience, the ways of managing symptoms, and better understanding and experience in EOL care. All in all, the variation in the use of PS worldwide may be due to differences in experience to use sedatives, different cultural aspects, PS methods, and definitions (Beller et al. 2015; Elsayem et al. 2009; Hurst et al. 2018; Schildmann et al. 2022).

EAPC and ESMO Guidelines recommend discussing the option of PS when the patient still has capacity to undergo these discussions. It is also recommended that if the patient permits, family members should be involved in these conversations. When the patient is unable to make decisions, it is important to have the patientʹs legal representatives’ opinion. Guidelines state that in the absence of advanced directive or a patientʹs health-care representative, the patients should be provided with “standard of care” symptom relief, if need be, also including PS (Cherny 2014; Cherny and Radbruch 2009). In our study in 69.1 % of the patients who had midazolam there was a record of discussion in the patient’s files about the possibility to apply PS. Thus, almost one out of three of the patients with midazolam had no documentation of upfront conversation. On average, over the nine years of study, some 40 % of the conversations eventually lead to the use of midazolam with sedative intent. In a study from Italy (Ingravallo et al. 2019) in hospice patients the discussion was held with 51.8 % of patients or their families and of those patients 68.6 % were sedated. Due to the retrospective nature of our study, it is impossible to know whether there actually were more of these conversations but without any record in the patient files. Yet, those should be made and documented appropriately.

The monitoring of the depth of sedation is highly recommended (Cherny and Radbruch 2009) and there are many scales to use; for example Richmond agitation sedation scale (RASS) (Sessler et al. 2002),the RASS RASS-PAL (Bush et al. 2014), and Ramsay Sedation Scale (RSS) (Monreal-Carrillo et al. 2017). In our study there was no mention of the use of scales, but the same is also true in other studies as only a few of them have documented the assessment of the depth of sedation (Arantzamendi et al. 2021; Dieudonné Rahm et al. 2021) indicating a general need for improvement.

DNAR was made in 74.5 % of patients who had midazolam prior to death. In the earlier years of the study the amount of DNAR in the whole population was close to 0 % explaining the relatively small number of decisions. In the later years nearly all patients had a DNAR decision made. Palliative and/or EOL care decision was made in most of the patients; yet 16.0 % of patients who had midazolam with a clear sedative intent did not have neither of these decisions. The decisions were made less in those patients with a single dose of midazolam. Perhaps the dying phase of those patients’ cancer was not recognized as early as of those with continuous infusion or multiple doses?

According to recent studies the most common cancer types in patients receiving sedation are lung and colorectal cancer (Alessia et al. 2022; Arantzamendi et al. 2021; Ingravallo et al. 2019; Prado et al. 2018). This was also seen in our data, lung cancer being the most common and colorectal cancer the second most common cancer types with midazolam. The most common refractory symptoms leading to PS include dyspnea, delirium, and pain (Beller et al. 2015; Caraceni et al. 2012; Garetto et al. 2018; Tan et al. 2023). This too is in line with our data. Dyspnea was especially seen in patients with only one dose of midazolam and all in all the distribution of refractory symptoms was a bit different when considering different types of midazolam usage. These results indicate that physicians should be aware of these symptoms toward the end of patients’ life. Especially in units providing EOL care for lung cancer patients, there should be expertise to provide PS. Overall, all units with EOL patients should be prepared to provide sufficient symptom control when fast changes in patients condition might happen.

In a review by Arantzamendi et al. (Arantzamendi et al. 2021) the median time until death varied between studies from 24 to 75 h. In many studies, the median duration is approximately 25 h (Monreal-Carrillo et al. 2017; Prado et al. 2018; RHPD et al. 2016a), which is in line with our results when considering the patients with continuous midazolam infusion. In those patients who received single doses of midazolam, the median duration was shorter and if a single dose was given, it was given median of 30 minutes prior to death. There is little information about the use of single doses of midazolam. The short timing between one dose and death may be a sign of rapid worsening of symptoms, which acquired fast reaction to provide symptom relief. Perhaps in patients with a continuous infusion, the state of the disease was more foreseeable.

The age of patients receiving PS tends to be around 65 years (Alessia et al. 2022; Imai et al. 2018; Maeda et al. 2016; Van Deijck et al. 2016b). Similarly to our results in a recent review younger age was significantly associated with PS (Tan et al. 2023). It has been theorized that younger patients may have more aggressive diseases and may experience more pain, whereas older patients may have a reduced level of consciousness at EOL and therefore demanding less sedation (Tan et al. 2023).

Patients, who had had a visit at the palliative outpatient clinic, had significantly more often midazolam. It may be speculated that they also had more intense symptoms demanding specialist palliative care. In our previous study we saw that the palliative and EOL care decisions were done late, mainly during the last days of life (Tolppanen et al. 2022). There was a trend of making these decisions more often in patients eventually sedated, which may indicate that the dying phase of those patients’ illness was recognized better as a continuum of the symptoms.

## Limitations and strengths

There are some limitations to the study. Our data included only patients who died at an oncology ward in a university hospital, and we excluded patients who died elsewhere, and thus the results may not be generalizable. For example, our patients may have had more intense symptoms demanding in-patient care in a specialist care unit. Information about PS discussions may be inaccurate as due to the nature of the study we cannot know exactly how it was discussed. Due to the study’s retrospective nature, it doesn’t bring current information about the use of midazolam. We excluded other use of sedatives as in almost all cases midazolam was used and the focus of this study was on midazolam use.

The strengths of this study are its relatively large size and that it brings valuable information about the use of midazolam at EOL care in cancer patients in Finland.

## Conclusion

Among patients dying at the oncology ward 14.7 % of patients received midazolam with sedative intent. Only 4.7 % of the patients had a continuous infusion. Patients having midazolam were more likely to be younger and had had a visit at the palliative outpatient clinic. Discussion about PS were documented in about two-thirds of sedated patients and no scales were used to document the depth of sedation. In the future discussion on sedation should be more appropriately documented and the depth measured.

## Supporting information

10.1017/S1478951525100461.sm001 Tolppanen et al. supplementary material Tolppanen et al. supplementary material

## Data Availability

The data that support the findings of this study are available from the corresponding author (AT) upon reasonable request.

## Supplementary material

The supplementary material for this article can be found at https://doi.org/10.1017/S1478951525100461.
