# Supplementary material for: Refractory symptoms and end of life midazolam use in cancer patients, a single center experience
Source: Palliat Support Care. 2025 Aug 8;23:e138. doi: 10.1017/S1478951525100461 (PMC13166639; doi:10.1017/S1478951525100461)
Supplement: Tolppanen et al. supplementary material [file S1478951525100461sup001.docx]

**Supplementary Table 1.** Cancer types, entire study population and patients with midazolam.

| **Cancer type** | **All patients, n (%)** | **Patients with midazolam, n (%)** |
| --- | --- | --- |
| Lung | 157 (24.6 %) | 30 (31.9 %) |
| Breast | 93 (14.6 %) | 8 (8.5 %) |
| Colorectal | 68 (10.6 %) | 11 (11.7 %) |
| Pancreas | 67 (10.5 %) | 8 (8.5 %) |
| Prostate | 26 (4.1 %) | 4 (4.3 %) |
| Kidney | 25 (3.9 %) | 2 (2.1 %) |
| Biliary tract and gallbladder | 19 (3.0 %) | 1 (1.1 %) |
| Melanoma | 24 (3.8 %) | 3 (3.2 %) |
| Head & neck | 17 (2.7 %) | 4 (4.3 %) |
| Hepatocellular | 19 (3.0 %) | 1 (1.1 %) |
| Gastric | 21 (3.3 %) | 3 (3.2 %) |
| Esophageal | 15 (2.3 %) | 2 (2.1 %) |
| Lymphoma | 10 (1.6 %) | 1 (1.1 %) |
| Urothelial | 9 (1.4 %) | 1 (1.1 %) |
| Glioma | 7 (1.1 %) | 0 (0 %) |
| Unknown primary | 14 (2.2 %) | 3 (3.2 %) |
| Other cancer | 48 (7.5 %) | 12 (12.8 %) |
